# Mass spectrometry data from proteomics-based screening of immunoreactive proteins of fully virulent *Brucella* strains using sera from naturally infected animals

**DOI:** 10.1016/j.dib.2015.07.029

**Published:** 2015-07-31

**Authors:** Gamal Wareth, Falk Melzer, Christoph Weise, Heinrich Neubauer, Uwe Roesler, Jayaseelan Murugaiyan

**Affiliations:** a Institute of Animal Hygiene and Environmental Health, Centre for Infectious Medicine, Freie Universität Berlin, Berlin, Germany; b Friedrich-Loeffler-Institut, Federal Research Institute for Animal Health, Institute of Bacterial Infections and Zoonoses, Jena, Germany; c Institute for Chemistry and Biochemistry, Freie Universität Berlin, Berlin, Germany

## Abstract

Here, we provide the dataset associated with our research article on comprehensive screening of *Brucella* immunoreactive proteins using sera of naturally infected hosts published in Biochemical and Biophysical Research Communications Wareth et al., 2015 [1]. Whole-cell protein extracts were prepared from *Brucella abortus* and *Brucella melitensis*, separated using sodium dodecyl sulfate polyacrylamide gel electrophoresis (*SDS*-*PAGE*) and subsequently western blotting was carried out using sera from bovines (cows and buffaloes) and small ruminants (goats and sheep). The mass spectrometry proteomics data have been deposited to the ProteomeXchange Consortium (http://proteomecentral.proteomexchange.org) via the PRIDE partner repository [2] with the dataset identifiers PXD001270 and DOI:10.6019/PXD001270.

**Specifications table**

**Table:** 

Subject area	Biology
More specific subject area	Immunoreactive proteins of *Brucella abortus* and *Brucella melitensis*
Type of data	Raw and processed/analyzed mass spectrometry data
How data was acquired	Mass spectrometry (Ultraflex II TOF/TOF MS, Bruker Daltonics, Bremen, Germany) analysis of in-gel digested proteins
Data format	Raw data (.mzML), peak (.mgf), processed and analyzed Mascot search engine (.dat) and result (.dat-pride.xml.gz)
Experimental factors	a) *Fully virulent Brucella strains*: *B. abortus* isolated from an infected cow in Turkey and *B. melitensis* isolated from an infected sheep in China. b) *Sera from naturally infected hosts species* (cattle, buffalo, sheep and goat). c) Western blot- and MALDI TOF MS-based identification of immunoreactive proteins.
Experimental features	*Brucella* isolates were cultured and whole cell protein extracts were prepared. The proteins were then separated on SDS-PAGE and Western blotting was carried out using sera collected from naturally infected hosts (cattle, buffalo, sheep and goat). The protein bands that matched with the western blot signals were excised from SDS-PAGE, digested with trypsin and subjected to mass spectrometry-based protein identification via peptide mass fingerprints.
	The spectra were acquired using Flex Analysis software version 3.3 (Bruker Daltonics, Leipzig, Germany), processed using Flex analysis software version 3.3 and protein identification was carried out using Biotools 3.0 (Bruker Daltonics, Leipzig, Germany) software integrated MASCOT search engine. The search results were stored as .html, .dat and .mgf. The mass spectrometry proteomics data have been deposited to the ProteomeXchange Consortium via the EBI-PRIDE partner repository.
Data source location	Berlin, Germany
Data accessibility	Accessible at ProteomeXchange Consortium via the PRIDE partner repository with the dataset identifiers PXD001270 and DOI:10.6019/PXD001270.

*Value of the data*

• The data further validate the protein identification presented in Wareth et al. [1].
• Virulent bacterial species/strains and sera from naturally infected hosts were used for identification of host-specific immunodominant proteins of *Brucella*.
• A comprehensive study using two different bacterial strains and sera from four different naturally infected hosts.

**Data, experimental design, materials and methods**

Brucellosis, also known as “*undulant fever*”, “*Malta fever*” or “*Mediterranean fever*,” is primarily a disease caused by gram-negative bacteria of the genus *Brucella*. Among the 11 known species, *B. abortus* (preferred host: cows and buffaloes) and *B. melitensis* (goats and sheep) share remarkably similar genomes [3]. However, they have remarkably different protein expression profiles [4]. This dataset is associated with the research article aimed towards comprehensive identification of immunoreactive proteins in the field isolates of *B. abortus* and *B. melitensis* by using the antibodies present in the sera collected from their naturally infected host species.

## 1 Bacterial strains, antisera and ethics.

The following two strains from the culture collection of the Institute for Bacterial Infections and Zoonoses (IBIZ); Friedrich-Loeffler-Institut (FLI); Jena; Germany were used for this study:

a *B. abortus* field strain was originally isolated from cattle in Turkey;
a *B. melitensis* field strain was isolated from sheep in China.

The isolates were biochemically identified [5] and the results were further confirmed by real-time PCR as previously described [6].

A total of 24 sera samples representing three each of positive (naturally infected) and three negative samples from four different host species (cattle, buffaloes, sheep and goats) was used from the sample collections of FLI; Jena; Germany. The samples was diagnosed to be Brucella-negative or positive by the recommended tests [5].

FLI as a federal authority in Germany and OIE reference laboratory for brucellosis is involved in the diagnosis of brucellosis samples submitted nationally and internationally. According to the German law, ethical approval or special permission concerning animal care is not required for the use of sera sent to FLI laboratories for diagnosis. In addition, the use of samples collected during routine diagnosis from Egypt was approved by the ethical committee at the office of dean, Faculty of Veterinary Medicine, Benha University, Ministry of Higher Education, Egypt.

## 2 Cell culture, protein extraction and western blotting

The bacterial strains were cultured for 48 h in Tryptic Soy Broth (TSB) at 37 °C with shaking, harvested during the stationary growth phase by centrifugation and washed twice with phosphate buffer saline. The cells were then reconstituted with 80% ethanol, centrifuged and the resulting cell pellet was air-dried completely. The whole-cell protein extraction, separated using an SDS-polyacrylamide gel and western blotting was carried out as described [1,7,8]. Diluted host sera (1: 200 diluted bovines and 1:500 small ruminants) was used as primary antibody source while 1:1000 diluted peroxidase-conjugated antibodies (anti-bovine IgG (H&L) (Chicken), anti-sheep IgG (H&L) (Donkey) and anti-goat IgG (H&L) (Chicken)) (Biomol-Rockland, Hamburg, Germany) served as secondary antibodies. The TMB kit^TM^ (3,3′,5,5′-tetramethylbenzidine liquid substrate, Sigma-Aldrich, Steinheim, Germany) was used for signal detection.

## 3 MALDI TOF MS measurement and protein identification

The SDS-PAGE bands that matched with the blot signals were excised, destained, digested with trypsin and the resulting peptides were spotted onto a MALDI target plate using the dried-droplet technique and HCCA (α-Cyano-4-hydroxycinnamic acid, Sigma-Aldrich, Steinheim, Germany) matrix as described [1,7]. MALDI-TOF MS measurements were carried out using an Ultraflex II TOF/TOF device and its associated software (Bruker Daltonics, Bremen, Germany). The spectra were acquired in positive reflector mode using the AutoXecute option of the FlexControl software version 3.3. The Peptide Calibration Standard II (Bruker Daltonics, Bremen, Germany) was used for external calibration. The MS or peptide mass finger print (PMF) spectra in the m/z range of 1000–3500 were acquired with the following setting: Ion source 1: 25 kV, ion source 2: 21.60 kV, lens: 10.50 kV, reflector 1: 26.30 kV, reflector 2: 13,60 kV. The processing and peak picking of the spectra was carried out automatically using Flex analysis software version 3.3. Seven of the intensive peaks were chosen for MS/MS product ion spectra (LIFT spectra) measurements in the laser-induced dissociation mode with the following settings: Ion source 1: 8 kV, ion source 2: 7.20 kV, lens: 3.60 kV, reflector 1: 29.50 kV, reflector 2: 13.90 kV, lift 1: 19.00 kV, lift 2: 3.00 kV.

Protein identification was carried out using BioTools 3.0 (Bruker Daltonics, Bremen, Germany) by comparing spectra (combined MS and MS/MS) data through MASCOT (http://www.matrixscience.com) against all entries of NCBInr (GenBank) as described earlier [7]. The parameter settings were as follows: Trypsin digestion – up to one missed cleavage; fixed modifications – carbamidomethyl (C); variable modifications – oxidation (M); peptide tol.: ±100 ppm; MS/MS tol.: ±0.8 Da and peptide charge:+1.

## 4 Mass spectrometry dataset deposit

The MS proteomics data was deposited at the ProteomeXchange (PX) Consortium [2] via the PRIDE (PRoteomics IDEntifications) partner repository at the European Bioinformatics Institute (http://www.ebi.ac.uk/pride/). The recorded MALDI-MS files were converted into.mzXML files using CompassXport software (Bruker Daltonics, Bremen, Germany) and the MASCOT search results were exported as.dat and.mgf. The.dat files were converted into PRIDE XML file using the freely available open-source tool PRIDE Converter [9] that works on wizard like graphical steps to include Minimum Information About A Proteomics Experiment (MIAPE). The entries in each PRIDE XML file were further verified for correctness using the freely available tool PRIDE inspector, version 2.1.1 [10]. The ‘Complete’ submission work flow of the freely available PX Submission tool (version 2.1.0: http://www.proteomexchange.org/submission) was chosen and the raw data (.mzML), peak (.mgf), search (.dat) and result (.dat-pride.xml.gz) of each sample were submitted at the ProteomeXchange Consortium. The complete dataset is now freely accessible with the dataset identifier PXD001270 and DOI:10.6019/PXD001270.
